# Lignocellulosic biomass fermentation: a roadmap for *Candida famata* and *Ogataea polymorpha*

**DOI:** 10.1093/femsyr/foaf046

**Published:** 2025-08-28

**Authors:** Dominik Wojdyła, Roksolana Vasylyshyn, Alicja Najdecka, Justyna Ruchala

**Affiliations:** Faculty of Biotechnology, Medical College, University of Rzeszów, Ćwiklińskiej 2D, Rzeszów 35-601, Poland; The Doctoral School of the University of Rzeszów, University of Rzeszów, 35-959 Rzeszów, Poland; Faculty of Biotechnology, Medical College, University of Rzeszów, Ćwiklińskiej 2D, Rzeszów 35-601, Poland; Department of Molecular Genetics and Biotechnology, Institute of Cell Biology, NAS of Ukraine, Drahomanov Street, 14/16, Lviv 79005, Ukraine; Faculty of Biotechnology, Medical College, University of Rzeszów, Ćwiklińskiej 2D, Rzeszów 35-601, Poland; Faculty of Biotechnology, Medical College, University of Rzeszów, Ćwiklińskiej 2D, Rzeszów 35-601, Poland

**Keywords:** lignocellulosic fermentation, pentose sugar utilization, stress tolerance in yeasts, metabolic engineering

## Abstract

The global transition to renewable energy sources requires efficient microbial platforms capable of fermenting carbon sources present in lignocellulosic biomass. Conventional yeasts like *Saccharomyces cerevisiae* face critical limitations, particularly in pentose sugar utilization and inhibitor resistance. This review focuses on two emerging nonconventional yeasts, *Candida famata* and *Ogataea polymorpha*, which exhibit native or engineered capacities to overcome these bottlenecks. We present a comparative analysis of their stress tolerance, metabolic versatility, and recent advances in genetic engineering, adaptive laboratory evolution, and heterologous expression systems. Their ability to grow on a wide range of sugars, tolerate fermentation inhibitors, and operate under industrial conditions underscores their potential as microbial platforms for sustainable bioprocessing. Key challenges and future directions are discussed to guide further development.

## Teaser

This review highlights *Candida famata* and *Ogataea polymorpha* as promising nonconventional yeasts with enhanced lignocellulosic fermentation capabilities, stress tolerance, and engineering potential for sustainable bioenergy production.

## Introduction

The bioconversion of lignocellulosic biomass into valuable chemicals and fuels remains a major challenge due to the recalcitrant nature of plant-derived substrates and the presence of fermentation inhibitors. While *Saccharomyces cerevisiae* is widely used in industrial fermentation, its limitations in pentose utilization and inhibitor resistance restrict its efficiency on lignocellulosic hydrolysates. This has led to growing interest in nonconventional yeasts with enhanced metabolic flexibility and stress tolerance. Among these, *Candida famata* and *Ogataea polymorpha* have emerged as promising candidates due to their thermotolerance, native or engineered pentose metabolism, and robustness under harsh industrial conditions. This review focuses on recent advances in the use of these yeasts for lignocellulosic fermentation and their potential to serve as next-generation microbial platforms.

### Lignocellulosic biomass as feedstock for bioprocessing: challenges and opportunities

The rising demand for energy and depletion of fossil fuel reserves have accelerated the search for sustainable and climate-neutral alternatives. One of the most promising candidates is lignocellulosic biomass, a nonfood, CO_2_-neutral feedstock derived from agricultural residues, energy crops, or forest by-products (Segers et al. 2024). Its availability, renewability, and noncompetition with food sources make it an attractive substrate for biotechnological applications.

**Figure 1.**
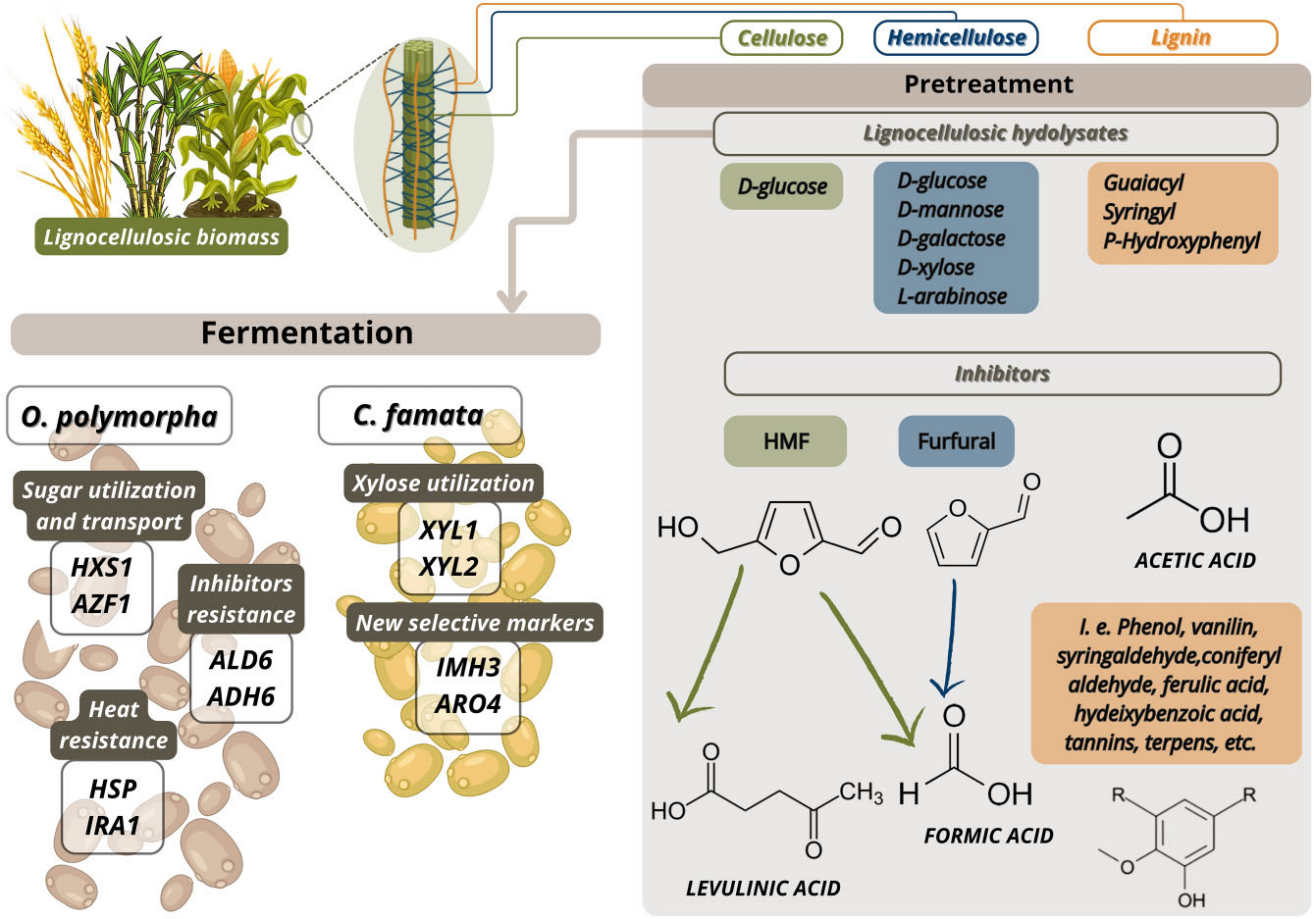
Metabolic and genetic engineering targets in *C. famata* and *O. polymorpha* relevant to lignocellulosic hydrolysate fermentation. Genetically modified strains show improved tolerance to inhibitors (e.g. furfural, HMF), thermotolerance, and pentose utilization. Modified from Tramontina et al. (2020).

Lignocellulosic biomass is primarily composed of three biopolymers: cellulose, hemicellulose, and lignin. Cellulose, a crystalline polysaccharide, provides mechanical strength; hemicellulose, an amorphous and branched heteropolysaccharide, binds the cellulose fibrils and contributes to cell wall integrity; and lignin, a complex aromatic polymer, acts as a structural matrix and barrier to enzymatic degradation (Ha et al. 1998, Ralph et al. 2019). While this architecture confers durability, it also poses major challenges to biomass deconstruction and conversion due to its chemical recalcitrance (Fig. 1).

Among these components, hemicellulose is the most amenable to hydrolysis, yielding fermentable sugars, such as xylose and arabinose. In contrast, lignin is highly resistant and hinders enzymatic access to cellulose. The lignin content varies significantly by biomass type, being lowest in agricultural residues and highest in conifers, affecting pretreatment requirements and conversion efficiency. Studies confirm that reducing lignin content improves yields in downstream processes such as biogas or ethanol production (Grabber 2005, Fernandes et al. 2009).

Despite its advantages, the use of lignocellulosic biomass is associated with several technological and economic barriers. These include the heterogeneity of feedstock composition, the formation of fermentation inhibitors, and potential contamination with toxic compounds. During thermochemical pretreatment, degradation of sugars and lignin releases inhibitory molecules, such as furfural, 5-hydroxymethylfurfural (HMF), organic acids, and phenolic compounds, which can impair microbial metabolism and reduce product yields (Renders et al. 2019, Ujor and Okonkwo 2022) (Fig. 1). In addition, biomass may contain trace amounts of heavy metals (e.g. from fertilizers, soil, or processing equipment) and synthetic residues, such as pesticides or plasticizers, particularly when derived from agricultural or municipal sources (Renders et al. 2019, Ujor and Okonkwo 2022). These contaminants can further compromise microbial performance and necessitate strain robustness.

Temperature plays a critical role in the efficiency of lignocellulosic ethanol production, particularly in processes that combine enzymatic hydrolysis and fermentation. Elevated temperatures not only enhance saccharification kinetics by increasing enzyme activity and substrate accessibility, but also enable simultaneous saccharification and fermentation (SSF), thereby reducing cooling demands and contamination risks in nonsterile industrial environments (Tramontina et al. 2020, Sengupta et al. 2022). Recent advances in emulsified SSF (eSSF) systems have further improved process performance by facilitating more efficient hydrolysis at high solids loading with reduced enzyme input (Hoffman et al. 2021). In this context, the thermotolerant yeast *O. polymorpha* has emerged as a promising host, demonstrating robust ethanol production at temperatures up to 50°C and outperforming mesophilic counterparts, such as *S. cerevisiae* and *Kluyveromyces marxianus* under thermal stress. Moreover, its application has been successfully extended to eSSF-based production of isobutanol, highlighting its versatility for both first- and next-generation biofuels. These findings underscore the importance of developing fermentation hosts that not only tolerate high temperatures but also retain productivity under inhibitory and multistressor conditions typical of lignocellulosic hydrolysates.

To address these limitations, recent metabolic and genetic engineering efforts have focused on improving the stress tolerance, pentose metabolism, and fermentation capacity of nonconventional yeasts, such as *O. polymorpha* and *C. famata*. These yeasts show considerable promise for lignocellulosic hydrolysate fermentation due to their innate thermotolerance and adaptability (Tramontina et al. 2020).

### Pretreatment and hydrolysis of lignocellulosic biomass

Lignocellulose pretreatment enables biomass conversion into fermentable sugars for biofuels and biochemicals. Effective pretreatment disrupts the lignin barriers, reduces cellulose crystallinity, and enhances the accessibility of enzymes, significantly improving sugar yields. Its efficiency depends on biomass type, process parameters (e.g. temperature, pressure, and pH), and the chemicals or microorganisms applied (Saha 2004, Kumar and Sharma 2017).

Various strategies—physical, chemical, physico-chemical, and biological are employed to alter the lignocellulosic matrix. Physical methods, such as milling or irradiation increase surface area and porosity, while chemical pretreatments using acids, alkalis, solvents, or green alternatives like ionic liquids and deep eutectic solvents help to solubilize hemicellulose and/or remove lignin products (Saha 2004, Kumar and Sharma 2017). Alkali pretreatment typically enhances enzymatic hydrolysis by delignification, whereas acid treatments target hemicellulose but may generate inhibitory by-products (Chong et al. 2025, Das et al. 2023, Saha 2004, Kumar and Sharma 2017).

Physico-chemical pretreatment combines mechanical and chemical actions. Techniques such as ammonia fiber expansion and soaking in aqueous ammonia enable delignification while preserving glucan and xylan (Das et al. 2023). Steam explosion, one of the most popular methods, uses high-pressure steam followed by rapid decompression to disrupt biomass structure and release hemicellulose. Liquid hot water utilizes high-temperature water to hydrolyze hemicellulose and increase cellulose accessibility (Kumar and Wyman 2009). Wet oxidation applies oxygen or hydrogen peroxide at high temperatures to oxidize lignin, whereas CO_2_ explosion uses supercritical CO_2_ to form carbonic acid under pressure, aiding in hemicellulose hydrolysis (Das et al. 2023, Kim and Lee 2007, Kumar and Wyman 2009, Ziegler-Devin et al. 2021).

Biological pretreatment utilizes microorganisms, especially white-rot fungi, which are highly effective in degrading lignin and enhancing enzymatic hydrolysis. Although all white-rot fungi possess lignin-degrading abilities, certain strains can selectively remove lignin while retaining high levels of cellulose, making them particularly valuable for efficient and targeted biomass processing (Blanchette 1991, Neshat et al. 2017). This method is eco-friendly, low-cost, and avoids the use of harsh chemicals (Chong et al. 2025). However, it is time-consuming and less efficient for industrial-scale applications (Blanchette 1991, Chong et al. 2025, Das et al. 2023, Neshat et al. 2017). While physical and chemical pretreatments are more efficient, biological methods offer eco-friendly alternatives, albeit with lower throughput (Das et al. 2023).

Combined pretreatment methods are increasingly explored to overcome the limitations of individual techniques. These hybrid methods aim to enhance efficiency while minimizing energy and chemical use. Overall, the choice of pretreatment depends on the type of biomass, desired sugar yield, cost considerations, and environmental impact. Continued research into novel and combined approaches, is essential for advancing sustainable biofuel production from lignocellulosic biomass (Das et al. 2023).

Lignin is a complex polymer that forms a resistant barrier in lignocellulosic biomass, preventing enzymatic access to cellulose and hemicellulose unless it is first modified or removed. To overcome this barrier, saprophytic fungi produce not only extracellular enzymes like cellulases, xylanases, and mannanases, but also a specialized oxidative lignin-degrading system known as Fenton’s reagents, which enables them to break down lignin and open phenyl rings. The soft-rot fungus *Trichoderma reesei*, along with its mutants, is extensively researched and utilized primarily for the industrial-scale production of cellulases and hemicellulases (Yang et al. 2014).

Cellulases and hemicellulases are essential for breaking down lignocellulose—cellulases convert cellulose to glucose, while hemicellulases hydrolyze hemicellulose into sugars such as xylose and arabinose (Zhang et al. 2012). These enzymes work synergistically to efficiently deconstruct the biomass and release fermentable sugars. However, their use faces challenges, including low stability, substrate inhibition, and poor efficiency in degrading recalcitrant biomass due to lignin interference and enzyme denaturation. Slow enzymatic activity also limits sugar yields (Nargotra et al. 2023, Østby et al. 2020). Advances in enzyme engineering, such as more stable variants, optimized enzyme cocktails, robust microbial sources, and improved reaction conditions, have enhanced hydrolysis efficiency (Nargotra et al. 2023). The resulting glucose and xylose are then utilized by engineered or naturally adapted microbial hosts, as detailed in the next sections. These improvements are key to making lignocellulose-based biofuels more cost-effective and sustainable (Nargotra et al. 2023, Østby et al. 2020, Xiao et al. 2024, Zhang et al. 2012).

### Inhibitors

During the pretreatment of lignocellulosic biomass, high temperatures and acidic or alkaline conditions degrade structural carbohydrates and lignin, resulting in the formation of inhibitory compounds, such as furfural, HMF, acetic acid, and a variety of phenolics. These compounds primarily originate from sugar dehydration and lignin fragmentation, and they significantly reduce microbial fermentation efficiency (Jönsson and Martín 2016). Furfural and HMF, classified as furan aldehydes, are among the most studied inhibitors. Furfural is generated by the acid-catalysed dehydration of C5 sugars (pentoses), primarily xylose, while HMF is derived from the dehydration of C6 sugars (hexoses), such as glucose and fructose (Almeida et al. 2009, Jönsson and Martín 2016). These compounds are highly toxic and exert their inhibitory effects mainly through the generation of reactive oxygen species (ROS), leading to oxidative stress. ROS can damage essential cellular macromolecules such as DNA, interfere with protein synthesis and folding, and disrupt membrane integrity, impairing transport and osmotic balance (Almeida et al. 2009).

The cellular response to this stress consumes significant energy, depleting ATP (adenosine triphosphate) and reducing NADH/NADPH (Nicotinamide Adenine Dinucleotide (reduced form) / Nicotinamide Adenine Dinucleotide Phosphate (reduced form)) pools, ultimately limiting growth and fermentation capacity (Kim and Hahn 2013). Additionally, furfural and HMF inhibit key metabolic enzymes, including those in the glycolytic pathway (Banerjee et al. 1981). Lignocellulosic hydrolysates also contain weak acids, particularly acetic acid and formic acid. Acetic acid is produced via the deacetylation of hemicelluloses, while formic acid arises from the degradation of furfural and HMF (Poontawee et al. 2017). The toxicity of these acids is primarily due to their uncoupling effect, which disrupts the mitochondrial proton gradient. Protonated weak acids can diffuse into the cytosol, dissociate, and acidify the intracellular environment. In response, the cell actively expels protons to restore pH homeostasis, consuming ATP and accumulating anions, which further compromises cell viability (Russell 1992).

Phenolic compounds, derived from lignin depolymerization, also pose substantial toxicity due to their membrane-disrupting effects and potential interference with enzyme function (Almeida et al. 2009, Miller et al. 2009). The combined presence of aldehydes, weak acids, and phenolics often leads to synergistic toxicity, which can disrupt fermentation or cause its complete failure. To mitigate these effects, two main strategies are commonly employed: (i) physical, chemical, or enzymatic detoxification of hydrolysates to reduce inhibitor levels, and (ii) the use of robust microorganisms that are naturally tolerant or have been engineered for resistance to inhibitors (Ravindran and Jaiswal 2016). However, detoxification approaches are often costly and may lead to sugar loss, making the development of inhibitor-tolerant strains a more sustainable solution (Alriksson et al. 2011). From the perspective of industrial biotechnology, the cytotoxic effects of inhibitors pose a major bottleneck, leading to decreased productivity, incomplete substrate utilization, and increased operational costs, ultimately compromising the economic feasibility of lignocellulosic bioprocessing (Horváth et al. 2003, Okonkwo et al. 2021).

### Microbial fermentation of lignocellulosic hydrolysates

#### Characteristics of a microbial host for lignocellulosic fermentation

As previously discussed, efficient valorization of lignocellulosic biomass requires not only the release of fermentable sugars through optimized pretreatment and hydrolysis strategies, but also microbial robustness against the inhibitory compounds inevitably formed during these processes. Since the formation of furans, phenolics, and organic acids is an inherent consequence of most physico-chemical pretreatments, fermentation hosts must combine efficient sugar metabolism with potent detoxification mechanisms, including aldehyde reductases, ABC transporters, and antioxidant systems (Fernández-Nino et al. 2018, Guo et al. 2022, Ujor and Okonkwo 2022). In addition, an ideal host should tolerate osmotic and thermal stress—both of which are commonly encountered under industrial conditions involving high solids loading and elevated fermentation temperatures (Avchar et al. 2022, Costa et al. 2014), and should preferably grow under low pH (Costa et al. 2014, Otieno et al. 2022) or oxygen-limited (Dekker et al. 2022) environments, which help reduce contamination risks and downstream processing costs (van Maris et al. 2006).

Beyond tolerance to stress factors, microorganisms intended for fermenting lignocellulosic hydrolysates must be able to efficiently utilize the full spectrum of available sugars. Industrial strains of *S. cerevisiae* do not naturally possess this ability. As a result, they are frequently genetically engineered by introducing genes encoding enzymes of the xylose utilization pathway, i.e. xylose isomerase or oxidoreductase, to enable efficient fermentation of this pentose (Kuyper et al. 2005, Matsushika et al. 2009). These yeast strains remain the dominant workhorse in industrial biotechnology due to their high ethanol productivity, GRAS (Generally Recognized As Safe) status, and well-established genetic toolkits, despite their inherent sensitivity to lignocellulose-derived inhibitors and organic acids (Brandt et al. 2021). Weak acids, such as acetic and formic acid, readily diffuse across the plasma membrane in their undissociated form and dissociate in the cytoplasm, lowering intracellular pH and disrupting metabolic homeostasis. To address this challenge, Wei et al. (2013) engineered *S. cerevisiae* strains capable of reducing acetic acid to ethanol under anaerobic conditions by integrating acetate reduction pathways with NADH regeneration derived from the xylulose utilization route. Recent strategies have expanded this approach by covalorizing acetate together with pentoses or xylo-oligosaccharides (XOS): Wang et al. (2023) demonstrated the use of acetate and formate as redox-balanced cosubstrates for free fatty acid production, while (Procópio et al. 2023) constructed *S. cerevisiae* strains capable of simultaneous intracellular XOS depolymerization and acetate fermentation, leading to increased ethanol yields and reduced xylitol formation under lignocellulosic hydrolysate conditions (Procópio et al. 2023).

In the case of furanic inhibitors such as furfural and HMF, detoxification is mediated by endogenous NAD(P)H-dependent reductases. Overexpression of *ADH6* and *ADH7*, which encode alcohol dehydrogenases with broad substrate specificity, enhances the yeast’s ability to convert these toxic aldehydes into less harmful alcohols. In parallel, *ZWF1*, encoding glucose-6-phosphate dehydrogenase, contributes to increased NADPH availability, thereby supporting reductive detoxification processes (Heer et al. 2009, Kim and Hahn 2013). Phenolic compounds, including vanillin, are among the most recalcitrant inhibitors in lignocellulosic hydrolysates. Vanillin, derived from the degradation of lignin, disrupts membrane integrity and induces oxidative stress by generating ROS. To improve resistance to phenolics, Ji et al. (2011) engineered *S. cerevisiae* to express a heterologous lacA gene from *Trametes* spp., which encodes a laccase capable of oxidizing and neutralizing extracellular phenolic compounds. Coexpression of *KAR2*, a chaperone protein involved in protein folding and secretion, improved the efficiency of laccase export, resulting in enhanced vanillin tolerance and increased conversion rates. Recent studies further emphasize the importance of global stress adaptation mechanisms. For example, adaptive laboratory evolution (ALE) applied to a xylose-fermenting *S. cerevisiae* strain enabled simultaneous tolerance to lignocellulosic inhibitors and insoluble solids, significantly improving ethanol yield under high-gravity fermentation. Transcriptomic analysis of the evolved strain revealed upregulation of genes associated with cell wall integrity (SRL1, CWP2, WSC2, and WSC4) and general stress responses (CDC5, DUN1, and GRE1), highlighting broader cellular strategies that underpin enhanced robustness (Moreno et al. 2022).

Unlike *S. cerevisiae, Scheffersomyces stipitis* (formerly *Pichia stipitis*) is a nonconventional yeast known for its innate ability to ferment xylose. Numerous studies have focused on enhancing fermentation efficiency and inhibitor tolerance to optimize bioethanol production by selected strains. A notable example is the hybrid strain SP2-18, obtained through recursive protoplast fusion between *S. cerevisiae* and *S. stipitis*, which demonstrated the ability to consume 34% of the xylose in the fermentation medium, achieving an ethanol productivity of 1.03 g/l/h, an ethanol yield of 0.447 g/g, and an ethanol concentration of 74.65 g/l—surpassing the parental *S. cerevisiae* strain in all parameters (Bajwa et al. 2011, Pereira et al. 2015, Tsegaye et al. 2024) (Table 1). Genome shuffling techniques have also been employed to improve tolerance to inhibitors present in various wood hydrolysates. As a result of these experiments, the modified strains were able to fully utilize glucose and xylose, producing ethanol concentrations ranging from 0.39% to 1.4% (w/v), whereas wild-type strains showed poor sugar utilization and only marginal ethanol production (Bajwa et al. 2011).

**Table 1.** Physiological and metabolic characteristics of selected yeasts applied in lignocellulosic ethanol and riboflavin production.

Ethanol fermentation										
Microorganism	Key features	Substrateversatility	Fermentationtemperature	Inhibitorresistance	Ethanoltolerance (v/v)	Ethanolproductivity (g/l/h)	Fermentationefficiency (g/g)	Geneticmodifications	Hydrolysate type	References
*S. cerevisiae*	Engineered for cofermentation of glucose and xylose; strong inhibitor tolerance	Glucose, xylose	35°C	High (tolerant to acetic acid and furans)	Up to 18%	2.24	0.485 (95.8% of theoretical)	Overexpression of *CCW12, ADH1, XylA*; evolved for hydrolysate tolerance	Synthetic mix + bagasse	Tsegaye et al. (2024)
*S . stipitis*	Evolved for xylose fermentation; moderate ethanol production; oxygen-limited performance	Xylose, glucose	30°C	Moderate (resistant to 20% hydrolysate)	~5%–6%	1.01	0.472	Adaptive evolution on rice straw hydrolysate; native XR/XDH pathway	Rice straw hydrolysate	Pereira et al. (2015)
*O. polymorpha*	Thermotolerant; native xylose metabolism; robust at high T; promising for industry	Glucose, xylose, methanol, nitrate	42°C–45°C	High	~6%–8%	Limited data	~0.35 (xylose)	Overexpression of *HXT1*; deletion of *TUP1, HAP4*; PPP flux optimization	Bagasse, xylose-rich hydrolysates	Kurylenko et al. (2018), Ruchala et al. (2017), Dmytruk et al. (2025), Vasylyshyn et al. (2020)
Riboflavin fermentation										
Microorganism	Key features	Substrate versatility	Fermentation temperature	Inhibitor resistance	Riboflavin productivity (g/l/h)		Riboflavin production per CDW (mg/g/CDW)	Genetic modifications	Hydrolysate type	References
*C. famata*	Pentose metabolism; riboflavin production; emerging ethanol platform	Glucose, xylose, arabinose	28°C–30°C	Moderate	~1.5		~182	Investigational (e.g. *XYL1/XYL2* expression)	Lignocellulosic hydrolysates	Dmytruk et al. (2011), Ruchala et al. (2022), Tsyrulnyk et al. (2020)

Beyond these well-characterized yeasts, recent research has focused on *C. famata* and *O. polymorpha*, which exhibit promising traits for lignocellulosic hydrolysate fermentation. *Candida famata* demonstrates potential for pentose metabolism (Dzanaeva et al. 2024), while *O. polymorpha* is known for its thermotolerance and ability to withstand high inhibitor concentrations (Ruchala et al. 2022, Vasylyshyn et al. 2025a). These microorganisms will be discussed in detail in a later section, as their unique metabolic properties offer new opportunities for improved bioconversion strategies. Future research should continue optimizing microbial hosts through metabolic engineering, adaptive evolution, and systems biology approaches to enhance sugar cofermentation, stress resistance, and product yield.

#### Case studies: C. famata and O. polymorpha

Considering the significant limitations of *S. cerevisiae* and the promising data for *S. stipitis*, other nonconventional yeast species are attracting the attention of researchers due to their unique physiological traits, which provide them with industrial utility. Among these, *C. famata* and *O. polymorpha* represent distinct and valuable platforms.

Among the leading riboflavin (vitamin B2) producers, *C. famata* stands out due to its ability to grow in low-pH environments and utilize a broad spectrum of substrates. Through the use of genetic engineering techniques, including the overexpression of genes involved in the purine biosynthesis pathway, production of riboflavin has been significantly enhanced (Dmytruk et al. 2011, Ruchala et al. 2022)

*Ogataea polymorpha* is characterized by heat tolerance, methylotrophic metabolism, and the ability to produce heterologous proteins at elevated temperatures. Recent studies have demonstrated its ability to grow at temperatures up to 50°C, making it one of the most thermotolerant eukaryotic microorganisms. Moreover, *O. polymorpha* can ferment xylose to ethanol and assimilate nitrate, distinguishing it from other methylotrophic yeasts. Advances in metabolic engineering have enabled the production of valuable chemicals, such as lactate, from methanol using modified strains of *O. polymorpha* (Liebal et al. 2021, Wefelmeier et al. 2023)

##### Candida famata (Debaryomyces subglobosus)

*Candida* yeasts excel in bioprocesses due to stress resistance and growth on cheap substrates. Some strains can also adapt to high osmotic stress caused by high concentrations of sugars or salts, which makes them particularly useful in biotechnology. The biotechnological potential of *Candida* species, especially those belonging to the CTG clade, is enormous in the era of synthetic biology. With their increased protein diversity and enhanced ability to adapt to changing environmental conditions, these yeasts are becoming extremely valuable tools in metabolic engineering and biochemical production. The CTG clade includes fungal species that exhibit a nonstandard genetic code—characterized by a change in the CTG codon from leucine to serine—and occurs in at least 75 species. This shift boosts protein diversity and adaptation (Krassowski et al. 2018). This unique genetic feature of the CTG clade—the reassignment of the CUG codon from leucine to serine—results in positions within proteins that normally contain leucine being occupied by either serine or leucine. In practice, this means that a single gene can produce heterogeneous protein variants differing in their physico-chemical and functional properties. This natural “dual identity” in protein composition generates increased proteomic diversity, which is invaluable for adaptation to variable and often stressful environmental conditions. Such proteomic flexibility can influence protein stability, interactions, and enzymatic efficiency, enabling yeasts to better adapt to harsh conditions such as high osmotic stress or the presence of toxins (Mühlhausen et al. 2021) *Candida* species can produce valuable metabolites like proteins, citric acid, xylitol, and xylose reductase. They were also historically used for riboflavin production. It should be noted that the classification of *Candida* species has been revised in recent years, with many species moving to other groups based on the analysis of various molecular markers. The MycoBank database, with synonyms and reclassified species, enables consistent comparison of new and past data (Daniel et al. 2014).

Despite the recent taxonomic reclassification of *C. famata* as *Debaryomyces subglobosus*, it remains relevant to consider this species within the context of the genus *Candida*, particularly when reviewing historical literature. A substantial body of earlier studies—especially those addressing its metabolic potential and genetic characteristics—identifies the organism as *C. famata*, which is still reflected in many publications, including those from our own research group (Dmytruk and Sibirny 2012). *Candida famata* (teleomorph *D. subglobosus*) is one of the most effective producers of riboflavin, also known as vitamin B2. Riboflavin is a precursor of flavin mononucleotide (FMN) and flavin adenine dinucleotide (FAD), essential coenzymes in energy metabolism and redox reactions in aerobic organisms. Due to its versatility and high market value, riboflavin is widely used in food, feed, pharma, and biotech industries. To enhance strain productivity and fermentation efficiency, main strategies focus on introducing extra copies of genes involved in riboflavin biosynthesis, its precursors, or regulatory elements. Without a fully sequenced *C. famata* genome, *Debaryomyces hansenii* gene sequences were used due to their high similarity. However, many details of riboflavin synthesis and regulation remain unclear and need further study (Sibirny 2023). The development of effective genetic modification tools and the application of advanced genetic engineering strategies proved crucial for enabling the effective use of nonconventional yeasts, thus opening up new possibilities for their future application in industrial biotechnology, also related to the processing of lignocellulosic biomass.

The basis was significant progress made in improving *C. famata* transformation methods by using established selectable markers and developing a new system involving the *LEU2* gene, which enables the generation and selection of leucine-auxotrophic strains. Specifically, *C. famata* mutants deficient in leucine biosynthesis (*leu2*) were isolated from a wild-type strain (VKM Y-9) and used as recipient strains in transformation experiments. The *LEU2* gene from *S. cerevisiae* was employed as a selectable marker to complement the auxotrophy. Transformants were identified based on their restored ability to grow in leucine-deficient medium, indicating successful integration or episomal maintenance of the introduced plasmid (Voronovsky et al. 2002). The unique CTG codon usage in *C. famata* required codon optimization of resistance genes from *D. hansenii*, enabling functional markers like *IMH3* and *ARO4* (Bratiichuk et al. 2020, Dmytruk et al. 2011a). The *Aspergillus terreus* blasticidin resistance gene was also introduced, while the *S. aureus ble* gene worked without modification (Dmytruk et al. 2006). In addition to marker development, new molecular tools have been developed, including insertional mutagenesis and targeted gene deletion methods, greatly expanding the genetic engineering toolbox available for this unconventional yeast A reporter system based on *K. lactis LAC4* (β-galactosidase) was developed. Several promoters were evaluated for potential use in future genetic constructs. Among them, the promoter of the *TEF1* gene, which encodes a translational elongation factor, showed the highest activity and was identified as the most robust (Ishchuk et al. 2008). The focus was also placed on a crucial aspect, namely enhanced transport of riboflavin outside the cell. By introducing the *RFE* gene from *D. hansenii*, encoding riboflavin excretase (a homolog of the mammalian BCRP protein), an increased riboflavin production was achieved, with a 1.5-fold reduction in its intracellular concentration as a result of efficient excretion of the compound outside the cell (Mao et al. 2004, Tsyrulnyk et al. 2020) These genetic modifications also improved production of flavin cofactors FMN and FAD. Overexpression of the *FMN1* (riboflavin kinase) gene from *D. hansenii* greatly increased riboflavin kinase activity and FMN levels (Fedorovych et al. 2023). Further optimization of culture conditions and overexpression of the *FAD1* (FAD synthetase) gene enhanced FAD accumulation compared to the parental strain (Yatsyshyn et al. 2014).

*Candida famata* demonstrates the ability to grow on a variety of sugars, including lactose, due to its *β*-galactosidase activity. This property has enabled the efficient production of riboflavin from whey waste, a by-product of the dairy industry, achieving a notable yield of 2.5 g/l (Ruchala et al. 2022). These findings highlight the potential of using industrial waste streams rich in fermentable sugars as substrates for the biosynthesis of value-added compounds. Given this metabolic flexibility, it is reasonable to explore the use of *C. famata* for riboflavin production from other complex feedstocks such as lignocellulosic biomass. This type of biomass is an abundant and underutilized source of fermentable sugars. However, the capacity of *Candida* species to utilize pentose sugars, particularly xylose, varies significantly across strains and species. While most *Candida* species are not known for robust xylose metabolism, certain nonconventional yeasts within the genus have shown promising potential. For instance, *C. tropicalis* and *C. magnoliae* have been successfully employed for xylitol production, and *C. glycerinogenes* has been studied for geraniol biosynthesis under controlled conditions (Kumar et al. 2022, Zhao et al. 2023). Despite these advances, bioconversion processes using lignocellulosic hydrolysates generally yield lower product titers than those using pure sugars like glucose. This is largely due to the limited capacity of many yeast strains to effectively metabolize the diverse sugar profile, particularly pentoses, in such hydrolysates. Although genetic engineering and other technological approaches have led to the development of *S. cerevisiae* mutants capable of more efficient xylose utilization, it is important to also consider nonconventional species that naturally ferment xylose efficiently, which could further enhance pentose fermentation processes (Cunha et al. 2020).

Based on these assumptions, it was demonstrated that *C. famata* can efficiently produce riboflavin using lignocellulosic waste as a substrate, specifically employing bagasse, a sugarcane residue in which xylose is the predominant pentose sugar. To enhance the efficiency of the bioprocess, a genetically improved strain known as BRPI (Best Riboflavin Producer Improved) was used (Dmytruk et al. 2020). This strain was engineered to overexpress the key genes involved in xylose metabolism—XYL1 and XYL2, which encode xylose reductase and xylitol dehydrogenase, respectively. These enzymes are essential for the conversion of xylose to xylulose, a key intermediate in the pentose phosphate pathway, thereby facilitating more efficient carbon flux toward riboflavin biosynthesis (Dzanaeva et al. 2024, Zha et al. 2014). The metabolic engineering of the BRPI strain resulted in a robust and stable phenotype capable of producing riboflavin at concentrations exceeding 1.5 g/l. The bioprocess was conducted under controlled conditions in lab-scale bioreactors, allowing precise monitoring and regulation of parameters, such as pH, temperature, aeration, and agitation, all of which are critical for optimal production yields. These experiments laid the foundation for future scale-up, as subsequent studies plan to transfer the process to larger, industrial-scale bioreactors (Dzanaeva et al. 2024).

Industrial agro-chemicals are often rich in pectin due to the processing of citrus fruits and vegetables (mainly sugar beet), and the main substances present in the hydrolysates obtained from them are l-arabinose, d-galactose, and d-galacturonic acid (Martins et al. 2020). The huge amounts of waste generated from sugar processing could be an excellent raw material for the production of new compounds in the future. However, the substances contained in them are not easily used by most yeasts. Interestingly, as has been shown, *C. famata* has an innate ability to use and decompose l-arabinose while simultaneously producing riboflavin. This metabolism supports survival in harsh, nutrient-limited conditions (Dzanaeva et al. 2024).

In the production of riboflavin from lignocellulosic hydrolysates, the type and amount of inhibitors present in these substrates have a key impact on the efficiency of the process. In order to minimize the negative impact of inhibitors on yeast, a number of strategies are used, such as metabolic modifications, the use of detoxification systems, or optimization of the fermentation process, but the approach based on ALE is particularly promising. This relies on selection schemes to enrich adapted genotypes, and the proliferation of cells with a better phenotype for a given environment favors their growth over poorly adapted cells, and these phenotypes, in turn, are the result of mutation (Barrick and Lenski 2013). Reverse engineering after ALE mutagenesis on lignocellulosic hydrolysates allows for precise identification of the molecular basis of yeast adaptation to a toxic environment containing inhibitors. Thanks to this approach, it is possible to identify key genes responsible for detoxification, improve the metabolism of utilization of various sugars by better understanding their pathway, or or guide future engineering strategies.

This method helped improve resistance to many industrial stresses in many microorganisms (Dragosits and Mattanovich 2013). Several genes have been identified as central to inhibitor resistance, including *ADH6, ALD6, GRE2*, and *ZWF1*, which are involved in enzymatic detoxification of furfural, HMF, and acetic acid. Transcription factors such as *YAP1* regulate oxidative stress response, while efflux pumps like *ATR1* and *PDR5* help expel toxic compounds from the cell. Additionally, *TAL1*, encoding transaldolase in the pentose phosphate pathway, contributes to increased NADPH regeneration, supporting redox balance and aldehyde detoxification. Genes such as *PEX5*, involved in peroxisomal protein import and oxidative stress mitigation, and *PEP3*, associated with vacuolar transport and detoxification processes, have also been linked to enhanced tolerance to lignocellulose-derived inhibitors (Menegon et al. 2022). Studies on nonconventional yeasts suggest that their adaptation mechanisms to stressors may share similarities with those observed in *S. cerevisiae*. Building on promising preliminary results—such as increased resistance to inhibitors and improved fermentation efficiency—further research is warranted to optimize adaptation strategies for *C. famata* in the fermentation of lignocellulosic hydrolysates. These findings indicate significant potential for the biotechnological application of *C. famata* in lignocellulose-based processes, and underscore the need for continued investigation into its metabolic adaptation and process integration.

##### Ogataea polymorpha

*Ogataea polymorpha* is a nonconventional yeast capable of utilizing methanol as the sole source of carbon and energy. Its unique physiological features—including high-density fermentation, broad substrate utilization (glucose, xylose, and methanol), and exceptional thermotolerance—make it a promising candidate for industrial biotechnology applications (Gao et al. 2023, Lehnen et al. 2019, Thorwall et al. 2020). These attributes have also been confirmed by screening strains from our internal strain collection (Ishchuk et al. 2009, Kurylenko et al. 2018, Ruchala et al. 2017, Vasylyshyn et al. 2020). For decades, *O. polymorpha* has served as a platform for recombinant protein production (Manfrão-Netto et al. 2019), yet its utility in lignocellulosic fermentation remains comparatively underexplored.

Despite the rise of high-throughput omics technologies facilitating insights into *O. polymorpha* metabolism (Patra et al. 2021), genomic studies are still limited. Only a few strains—DL-1, CBS4732, and NCYC 495–have undergone full genome sequencing (Ramezani-Rad et al. 2003, Riley et al. 2016, Suh and Zhou 2010). Nevertheless, this yeast is emerging as a viable chassis for future biotechnological applications, especially in the context of lignocellulosic biomass valorization.

One of its key advantages is the native ability to metabolize xylose, one of the principal sugars in lignocellulosic hydrolysates, via a NAD(P)H-dependent reductive pathway involving XYL1 (xylose reductase), XYL2 (xylitol dehydrogenase), and XYL3 (xylulokinase) (Kurylenko et al. 2021, Ruchala et al. 2017). Carbon-responsive regulators like MIG1 and CAT8 govern this pathway through glucose-dependent repression and activation (Kurylenko et al. 2021, Ruchala et al. 2017). The XR–XDH route is prone to redox imbalance due to cofactor mismatch, XYL1 prefers NADPH, while XYL2 uses NAD⁺, which often results in xylitol accumulation and reduced ethanol yields. To address this, metabolic engineering strategies have focused on cofactor rebalancing (e.g. NADH-preferring XYL1 variants) and the introduction of heterologous xylose isomerase pathways that bypass the redox constraint entirely (Gao et al. 2023).

In addition to its native xylose metabolism, *O. polymorpha* exhibits regulatory elements for sugar sensing and transport. Recent studies have implicated *HXS1* and *AZF1* in glucose/xylose sensing and uptake, suggesting a broader regulatory network involved in carbon source adaptation (Semkiv et al. 2022). While *O. polymorpha* naturally utilizes glucose, xylose, and methanol, the efficient fermentation of other sugars commonly found in lignocellulosic hydrolysates, such as arabinose and cellobiose, typically requires metabolic engineering. For example, arabinose utilization likely relies on native but weakly expressed pathways involving l-arabinose reductase and l-arabinitol dehydrogenase, leading to xylulose-5-phosphate via l-xylulose intermediates (Ruchala and Sibirny 2021). Improved cellobiose fermentation, in turn, has been achieved by introducing heterologous transporters and hydrolases. Evolved strains demonstrate enhanced fermentation of cellobiose and mixed sugars at elevated temperatures, leveraging both native and engineered pathways, including xylose isomerases from *Piromyces* and *L. phytofermentans* (Gao et al. 2023, Vasylyshyn et al. 2024). Cellobiose, a disaccharide derived from cellulose hydrolysis, along with pentoses such as xylose and arabinose, represents a key component of lignocellulosic hydrolysates that must be efficiently utilized for effective bioconversion. These results collectively highlight *O. polymorpha* as a robust and versatile platform for lignocellulose-derived bioproducts (Das et al. 2023, Gao et al. 2023, Vasylyshyn et al. 2024).

Compounds such as furfural and weak acids, generated during biomass pretreatment, interfere with protein folding and disturb redox balance. In response, *O. polymorpha* activates protective mechanisms involving heat-shock proteins (Hsps) and trehalose accumulation, which collectively improve tolerance to combined thermal and chemical stressors (Choo et al. 2021). The heat-shock response (HSR) in *O. polymorpha* is typically induced at 47°C–49°C and is regulated by heat shock transcription factors (Hsfs), which mediate the expression of molecular chaperones such as Hsp70 (Barna et al. 2018). Interestingly, deletion of *HSP70* enhances thermotolerance, whereas its overexpression impairs cell viability (Titorenko et al. 1996). This paradoxical behavior suggests that excessive Hsp70 levels may interfere with cellular homeostasis, possibly by sequestering unfolded proteins or delaying proteolysis, thereby exacerbating stress rather than alleviating it. These findings indicate that the function of Hsp70 is dose-dependent and tightly regulated (Barna et al. 2018). In contrast, overexpression of *HSP16* and *HSP104* confers increased heat resistance and supports efficient fermentation at elevated temperatures (Ishchuk et al. 2009). Mutational analysis of *Ophsf1* further revealed noncanonical regulation of HSR in *O. polymorpha* compared to *S. cerevisiae*, with domain-specific contributions to stress adaptation (Choo et al. 2021). Collectively, these data highlight the coordinated roles of both small Hsps and major chaperones in mediating resilience to industrial fermentation conditions. While direct evidence remains limited, it is plausible that overlapping protective mechanisms underlie the robustness of *O. polymorpha* under multifactorial stress (Mira et al. 2010, Stanley et al. 2010).

Our recent research focuses on the IRA1 gene, a key regulator of thermotolerance in *O. polymorpha*. We found that overexpression of *IRA1* enables growth at elevated temperatures up to 47°C, with particularly strong effects observed when cells are grown on l-arabinose and xylose as carbon sources (Vasylyshyn et al. 2025b). This indicates that the thermal stress response in *O. polymorpha* is influenced not only by temperature itself but also by the specific type of sugar metabolized. The *IRA1* gene likely contributes to thermotolerance through modulation of the Ras-cAMP signaling pathway, a key regulator of stress responses and carbon metabolism. Our findings suggest a sugar-specific influence on this pathway, possibly via altered fluxes through NAD(P)H-dependent reactions and oxidative stress buffering, reinforcing the link between carbon source utilization and heat resilience. In other words, the way the yeast processes different carbon sources can modulate its ability to withstand heat stress. This insight is especially important for industrial fermentation of lignocellulosic hydrolysates, which contain a complex mixture of sugars, including pentoses like xylose and arabinose, and where high temperature tolerance is critical for efficient bioprocessing. These findings suggest that the thermotolerance of *O. polymorpha* is not merely a passive trait but is actively modulated by carbon metabolism, likely via cross-talk between sugar catabolism, redox balancing, and stress-responsive signaling pathways. For instance, differential utilization of pentoses such as arabinose and xylose may affect intracellular NAD(P)H availability, influencing the oxidative stress response under high-temperature conditions (Vasylyshyn et al. 2025b).

While previous work has tackled tolerance to individual inhibitors in hydrolysates, studies using undetoxified lignocellulosic substrates remain rare (Martínez-Cartas et al. 2024, Ni et al. 2025). ALE of high-performing strains improved growth in undiluted bagasse hydrolysates at 45°C–47°C (Ruchala et al. 2025, unpublished). Still, cane straw hydrolysate remained a major challenge due to its high inhibitory load. Here, ALD6 overexpression enhanced ethanol yields and biomass accumulation, likely due to its role in detoxifying furans and supporting redox balance, whereas ADH6 had no measurable impact (Ruchala et al. 2025, unpublished). This distinction underscores the necessity of pathway-specific interventions when engineering tolerance to complex inhibitory environments.

In summary, *O. polymorpha* offers a potent yet underutilized platform for lignocellulosic bioprocessing. Its innate ability to metabolize both hexose and pentose sugars (including xylose and arabinose), tolerate elevated temperatures, and withstand inhibitory compounds without extensive detoxification gives it a competitive edge over conventional hosts like *S. cerevisiae*. These properties make *O. polymorpha* an excellent candidate for consolidated bioprocessing (CBP) approaches, where saccharification and fermentation occur in a single step. Fully harnessing this potential will require integration of omics-guided engineering (e.g. transcriptomic analysis), targeted stress pathway modulation (e.g. Hsf1 and *IRA1* manipulation), and adaptive evolution strategies tailored to undetoxified hydrolysates—an approach that may accelerate the development of robust CBP-ready strains.

### Genetic engineering prospects in emerging yeast platforms

Efficient genetic tools facilitate the construction of robust microbial cell factories (Cai et al. 2019, Dmytruk and Sibirny 2012, Huang et al. 2023). Recent advances in synthetic biology have substantially expanded the genetic toolkit for *O. polymorpha*, facilitating its development as a microbial cell factory. Efficient gene targeting strategies, including the use of an NHEJ-deficient *yku80* mutant and PCR-based deletion methods, have improved HR and enabled precise genome editing (Saraya et al. 2012). In parallel, extensive characterization of regulatory elements has led to the identification of a broad spectrum of promoters—such as strong constitutive promoters (*GCW14, PDH, GPI*, and *TEF1*), inducible promoters (*ICL1, LRA3*, and *LRA4*), and methanol-responsive promoters (*FDH, FLD, TAL1, CAT*, and *DAS*)—providing tunable control over gene expression across various carbon sources (Yan et al. 2022, Zhai et al. 2021). Moreover, terminators like *MOX, CAT, FMD, PMA1*, and heterologous elements such as *AOX1* from *P. pastoris* and *TEF1* from *Aphis gossypii* have been shown to significantly influence mRNA stability and transcript levels, enabling up to 6-fold modulation of gene expression independently of the promoter used (Wefelmeier et al. 2022). Together, these elements offer a modular, mix-and-match system for fine-tuning genetic constructs, streamlining metabolic pathway optimization in *O. polymorpha*.

A range of genetic engineering tools has been developed for *C. famata* and *O. polymorpha*, expanding their utility in metabolic engineering. For *C. famata*, a promoter assay system has been established and several strong promoters have been cloned (Ishchuk et al. 2008). In parallel, new dominant selection markers such as the *BSD* gene from *A. terreus* (encoding blasticidin S deaminase), the *AUR1* gene from *O. polymorpha*, and *IMH3* (IMP dehydrogenase) have been validated for transformation and selection in both yeasts (Bratiichuk et al. 2020). Notably, *IMH3* enabled the construction of recombinant *O. polymorpha* strains overexpressing *TAL1, TKL1*, and *AOX1*, leading to improved ethanol production from xylose. Meanwhile, *BSD* served as an efficient marker in *C. famata*.

Efficient transformation protocols, including electroporation and the lithium, acetate method (Heo et al. 2003, Sibirny and Voronovsky 2009, Voronovsky et al. 2002), along with strategies for integrating large synthetic constructs (>10 kb), such as multigene expression cassettes, have supported the stable integration of complex metabolic pathways (Wagner and Alper 2016).

In *O. polymorpha*, recent advances in CRISPR/Cas-based genome editing have significantly accelerated strain development. Early CRISPR/Cas9 platforms demonstrated efficient marker-free genome editing and pathway optimization (Juergens et al. 2018, Numamoto et al. 2017, Wang et al. 2018). To address the species’ inherently low homologous recombination (HR) efficiency (Saraya et al. 2012), recombination machinery engineering strategies have been introduced. These include the overexpression of HR-related genes and suppression of the NHEJ (Non-Homologous End Joining) pathway, leading to increased HR efficiency (60%–70%) and enabling complex genome rearrangements, such as *in vivo* assembly of multigene pathways for fatty alcohol biosynthesis (Gao et al. 2021).

Further refinements include the development of a tRNA–sgRNA fusion system for efficient Cas9 targeting (Numamoto et al. 2017) and the recent adaptation of the CRISPR-Cas12a (Cpf1) system for multiplex editing, allowing simultaneous modulation of multiple loci (Hou et al. 2025). Additionally, several neutral integration sites have been identified for stable heterologous expression without disrupting native gene function (Yu et al. 2021). A genome-scale metabolic model has also been established for *O. polymorpha*, enabling rational strain design (Liebal et al. 2021).

In contrast, genome editing tools in *C. famata* remain underdeveloped. Although this yeast shares high ORF (Open Reading Frame) sequence identity with *D. hansenii* (Ishchuk et al. 2008), differences in regulatory regions and the lack of a complete genome sequence still constrain its biotechnological exploitation. Given that *C. famata* represents the asexual (anamorphic) stage of *D. hansenii* (Kurtzman and Robnett 2013), genome engineering strategies developed for *D. hansenii*, including recently established CRISPR/Cas9-based markerless editing systems with up to 95% efficiency (Spasskaya et al. 2021), may be transferable to *C. famata*. Although no CRISPR-based protocols have yet been reported in *C. famata*, ongoing progress in transformation methods and cross-species tool adaptation (Tsyrulnyk et al. 2020) lays the groundwork for future development.

The development of efficient genetic tools is essential for advancing microbial cell factory construction, especially for the production of valuable compounds from lignocellulosic hydrolysates. Despite significant progress in genetic engineering for *C. famata* and *O. polymorpha*, the major challenge remains the presence of toxic compounds in hydrolysates and the complexity of fermentation processes. These factors complicate the optimization of fermentation conditions and strain design for enhanced product yield. Nonetheless, modern advancements in genetic engineering, including the development of CRISPR/Cas9-based genome editing systems and improved transformation protocols, have significantly advanced our ability to manipulate these yeast species. However, addressing the challenges posed by the toxic by-products of hydrolysis and optimizing fermentation remain critical hurdles for fully realizing the potential of *C. famata* and *O. polymorpha* in biotechnology applications.

## Conclusions

This MiniReview presents a focused and timely overview of the application of *C. famata* and *O. polymorpha* in lignocellulosic bioprocessing, addressing a gap in the literature where most reviews cover general challenges of lignocellulosic fermentation but rarely explore the specific biotechnological potential of these two nonconventional yeasts.

The novelty of this work lies in its organism-centered comparative analysis, highlighting recent genetic engineering strategies, such as CRISPR/Cas9, adaptive evolution, and transporter optimization tailored to these species. Additionally, it emphasizes progress in fermenting real lignocellulosic hydrolysates rather than relying solely on synthetic media, while also demonstrating the capacity of these yeasts to produce valuable compounds like riboflavin and ethanol under stress conditions relevant to industrial biorefineries.

By integrating physiological insights with applied metabolic engineering, this MiniReview aims to provide a clear roadmap for developing *C. famata* and *O. polymorpha* as next-generation microbial platforms, guiding researchers toward promising and underexplored opportunities in lignocellulosic biomass valorization.
